# Diversity and Bioactive Potential of Actinobacteria from Unexplored Regions of Western Ghats, India

**DOI:** 10.3390/microorganisms8020225

**Published:** 2020-02-07

**Authors:** Saket Siddharth, Ravishankar Rai Vittal, Joachim Wink, Michael Steinert

**Affiliations:** 1 Department of Studies in Microbiology, University of Mysore, Manasagangotri, Mysore 570 006, India; saketsiddharth@gmail.com; 2 Microbial Strain Collection, Helmholtz Centre for Infection Research GmbH (HZI), Inhoffenstrasse 7, 38124 Braunschweig, Germany; joachim.wink@helmholtz-hzi.de; 3 Institute of Microbiology, Technische Universität Braunschweig, 38106 Braunschweig, Germany; m.steinert@tu-bs.de

**Keywords:** Western Ghats, diversity, actinobacteria, antimicrobial, MRSA, antioxidants

## Abstract

The search for novel bioactive metabolites continues to be of much importance around the world for pharmaceutical, agricultural, and industrial applications. Actinobacteria constitute one of the extremely interesting groups of microorganisms widely used as important biological contributors for a wide range of novel secondary metabolites. This study focused on the assessment of antimicrobial and antioxidant activity of crude extracts of actinobacterial strains. Western Ghats of India represents unique regions of biologically diverse areas called “hot spots”. A total of 32 isolates were obtained from soil samples of different forest locations of Bisle Ghat and Virjapet situated in Western Ghats of Karnataka, India. The isolates were identified as species of *Streptomyces*, *Nocardiopsis*, and *Nocardioides* by cultural, morphological, and molecular studies. Based on preliminary screening, seven isolates were chosen for metabolites extraction and to determine antimicrobial activity qualitatively (disc diffusion method) and quantitatively (micro dilution method) and scavenging activity against DPPH (2,2-diphenyl-1-picrylhydrazyl) and ABTS (2,2′-Azino-bis(3-ethylbenzothiazoline-6-sulfonic acid) radicals. Crude extracts of all seven isolates exhibited fairly strong antibacterial activity towards MRSA strains (MRSA ATCC 33591, MRSA ATCC NR-46071, and MRSA ATCC 46171) with MIC varying from 15.62 to 125 μg/mL, whereas showed less inhibition potential towards Gram-negative bacteria *Salmonella typhi* (ATCC 25241) and *Escherichia coli* (ATCC 11775) with MIC of 125–500 μg/mL. The isolates namely S1A, SS5, SCA35, and SCA 11 inhibited *Fusarium moniliforme* (MTCC 6576) to a maximum extent with MIC ranging from 62.5 to 250 μg/mL. Crude extract of SCA 11 and SCA 13 exhibited potent scavenging activities against DPPH and ABTS radicals. The results from this study suggest that actinobacterial strains of Western Ghats are an excellent source of natural antimicrobial and antioxidant compounds. Further research investigations on purification, recovery, and structural characterization of the active compounds are to be carried out.

## 1. Introduction

The quest for novel biologically active secondary metabolites from microorganisms continues to rise due to emergence of drug resistance in pathogens causing life threatening diseases around the globe [1]. Particularly, methicillin resistant *Staphylococcus aureus* (MRSA) and methicillin resistant *Staphylococcus epidermidis* (MRSE) strains are not only exposed to hospital-acquired infections but also to community-acquired infections [2]. The mortality and morbidity associated with these infections are largely affecting economic conditions of patients and hospitals [3]. Therefore, there is an urgent need for developing novel and effective antimicrobial agents to overcome or delay acquired resistance to existing drugs. Reactive oxygen species (ROS) play an important role as signaling molecules involved in mitogenesis. However, high generation of ROS during aerobic metabolism creates oxidative stress within the intracellular milieu causing oxidative damage to cells [4]. The oxidative stress caused is often associated with many human diseases including cancer, diabetes [5], cardiovascular [6], and neurodegenerative diseases [7]. In order to withstand the oxidative stress caused, cells or organisms make use of antioxidants, that are able to block or delay the damage caused by several possible mechanisms such as halting chain reactions, preventing the formation of free radicals, neutralizing the singlet oxygen molecule, promoting anti-oxidant enzymes, and inhibiting pro-oxidative enzymes [8]. Formation of free radicals can be prevented by antioxidant systems present within the cells. However, these defense mechanisms are insufficient to prevent the damages that arise, therefore exogenous antioxidants through dietary intake and supplements are required [9]. Natural antioxidants are found abundantly in metabolites produced by microorganisms. These products have consistently been considered as mainstay for drugs with various interesting biological activities [10]. They are considered to be an excellent scaffold for the formulation and development of antibiotics, antioxidants, immunomodulators, enzyme inhibitors, anticancer agents, plant growth hormones, and insect control agents [11]. With many improved techniques under combinatorial chemistry for high throughput findings of novel compounds, natural products from microbial sources have been screened extensively and gained much attention owing to their massive chemical and biological diversity [12]. Under various screening strategies, the rate of discovery of natural products has increased many folds, of which around 22,250 bioactive compounds are of microbial origin [13]. Among microorganisms, actinomycetes have contributed nearly 45% of all the reported metabolites [14].

A major group of natural products from microbial origin have been identified from organisms that inhabit the soil. Since soil itself is a mixture of minerals and organic matter, the filamentous bacteria are predominantly more present in the gaps between the soil particles than their unicellular counterparts [15]. Actinobacteria are ubiquitous in soils. They are responsible for biodegradation and biodeterioration processes in nature. Their flexile and proven abilities have prompted biologists to screen these organisms from unexplored niche habitats in order to obtain novel molecules [16].

Western Ghats of India is considered as one of the global biodiversity hotspots covering an area of 180,000 km^2^ and harbors numerous species of plants, animals, and microbes [17]. The unique biodiversity of Western Ghats is conserved and protected by wildlife sanctuaries, national parks, and biosphere reserves situated in states where hill ranges run through, like Karnataka, Gujarat, Tamil Nadu, Maharashtra, and Kerala [18]. The forest regions in Western Ghats are largely underexplored, though in recent times few studies were carried out for bioprospection. Ganesan et al. [19] reported larvicidal, ovicidal, and repellent activities of *Streptomyces enissocaesilis* (S12–17) isolated from Western Ghats of Tamil Nadu, India. Actinobacterial strains isolated from Western Ghats soil of Tamil Nadu were reported to produce antimicrobial compounds against range of pathogens [20]. In the present study, the forest range in Western Ghats of Karnataka was studied for microbial population and taxonomical identification of potential actinobacteria. An attempt was also made to characterize microbial diversity for the potential to produce antioxidants and antimicrobial compounds.

## 2. Materials and Methods

### 2.1. Site, Sampling, Pre-Treatment, and Selective Isolation

Soil samples were collected from different forest locations of Bisle Ghat and Virajpet in Western Ghats regions of Karnataka, India. The samples were collected from a depth of 15–25 cm in dry sterile insulated containers and stored aseptically at 4 °C until subjected to plating. Samples were air dried in a hot air oven (Equitron, India) at 50 °C for 72 h. Pre-treated samples were ground aseptically with mortar and pestle and serially diluted up to 10^−6^ in 10-fold dilution. The aliquots of each dilution (100 μL) were spread evenly on starch casein agar (SCA, Himedia, India) plates in triplicates supplemented with cycloheximide (30 μg/mL) and nalidixic acid (25 μg/mL). The plates were incubated at 28 ± 2 °C for 14 days. Emerging colonies with different morphological characters were selected and the purified strains were maintained on International *Streptomyces* Project (ISP-2, Himedia, India) agar slants and stored at 4 °C as stock for further use.

### 2.2. Morphological Characterization of Isolates

Morphological characteristics of isolates were assessed by scanning electron microscopy (SEM). Bacterial colonies were inoculated in ISP-2 medium and incubated at 28 ± 2 °C for 7 days. Cells were centrifuged (Eppendorf, USA) at 8000× *g* for 10 min and pellet was resuspended in 2%–5% gluteraldehyde (Sigma, Burlington, VT, USA) prepared in 0.1M phosphate buffer, pH 7.2. After incubating samples for 30 min, supernatant was discarded and pellet was resuspended in 1% osmium tetraoxide (Sigma, Burlington, VT, USA), incubated for 1 h and centrifuged at 5000 × *g*. To the pellet, sterile water was added and centrifuged twice for 10 min at 5000 × *g*. For dehydration, the pellet was resuspended in 35% ethanol for 10 min, 50% ethanol for 10 min, 75% ethanol for 10 min, 95% ethanol for 10 min, and a final wash with 100% ethanol for 10 min. For SEM analysis, sterilized aluminum stubs and cover slips were inserted into the SCA plates at an angle of about 45 °C. The plates with stubs and coverslips were incubated at 37 °C for 24 h to check any contamination. After 24 h, isolates were introduced along the line where the surface of the stub met the agar medium and incubated at 28 ± 2 °C for 7 days. The stubs were then carefully removed and coated under vacuum, with a film of gold for 25–30 min and viewed on the scanning electron microscope (Zeiss Evo 40 EP, Germany). 

### 2.3. Molecular Identification and Phylogenetic Analysis

The total genomic DNA of bacteria was extracted by phenol-chloroform method, quality checked by agarose gel electrophoresis and quantified using NanoDrop1000 (Thermo-Scientific, USA). The PCR amplification of 16S rRNA gene was carried out with universal primers: 27F (5′-AGA GTT TGA TCC TGG CTC AG-3′) and 1492R (5′- ACG GCT ACC TTG TTA CGA CTT-3′) using the following conditions: initial denaturation temperature was set at 95 °C for 5 min, followed by 35 cycles at same temperature for 1 min, primer annealing at 54 °C for 1 min, and primer extension at 72 °C for 2 min. The reaction mixture was kept at 72 °C for 10 min subsequently and then cooled to 4 °C. The PCR products were checked in 1.5% agarose gel and visualized in a UV transilluminator (Tarsons, India) and the gel imaging was done using a Gel documentation system (Bio-Rad, USA). The amplified PCR products were sequenced using same set of primers (27F’ and 1492R’) on Applied Biosystems 3130 Genetic Analyzer (Applied Biosystems, USA). The genetic relationship between the strains was determined by neighbor-joining tree algorithm method. The phylogenetic tree was constructed with a bootstrapped database containing 1000 replicates in MEGA 7.0 software (Mega, Raynham, MA, USA). The nearly complete 16S rRNA consensus sequences were deposited in the GenBank database. 

### 2.4. Isolates Cultivation and Metabolites Extraction

Pure isolates were subcultured in Tryptone Yeast Extract broth as seed medium (ISP-1 medium, Himedia, India) at 28 °C for 2 weeks prior to fermentation process. The production medium, ISP-2 was autoclaved at 121 °C and 1.5 atm for 15 min. Fermentation was carried out in 750 mL of (in 7 nos. conical flasks −1000 mL) ISP-2 medium, shaking at 140 rev min^−1^ for 14 days at 28 °C, inoculated with 250 µL of seed medium. After incubation, the culture medium was split into mycelium and filtrate by centrifugation at 12,000× *g* for 15 min. The cell free supernatant from each flask was subjected to extraction thrice with equal volume of ethyl acetate (Qualigens Fine Chemicals Pvt. Ltd., San Diego, USA) and the organic phase was concentrated by rotary vacuum evaporator (Hahn-Shin, Bucheon, South Korea) at 50 °C. The crude concentrate was dried in a desiccator and suspended in methanol prior to bioactivity screening assays. 

### 2.5. Antimicrobial Susceptibility Test

#### 2.5.1. Disc Diffusion Assay

Antimicrobial susceptibility assay was carried out by disc diffusion method against methicillin-resistance *Staphylococcus aureus* (MRSA ATCC 33591, MRSA ATCC NR-46071 and MRSA ATCC 46171), Gram-negative bacteria (*Salmonella typhi* (ATCC 25241) and *Escherichia coli* (ATCC 11775)) and fungus (*Fusarium moniliforme* (MTCC 6576)). Gentamicin and Nystatin discs were used as positive control. The sterile discs (6mm, Himedia) were impregnated with 30 μL of crude extract dissolved in 0.5% DMSO. Discs impregnated with 0.5% DMSO (Qualigens Fine Chemicals Pvt. Ltd., San Diego, USA) were used as solvent control. The plates were left for 30 min at 4 °C to allow the diffusion of extracts before they were incubated for 24–48 h at 37 °C. The clear zones of inhibition observed around discs suggested antagonistic activity against test organisms and diameter of inhibition zones were measured subsequently. The test was performed in triplicate. 

#### 2.5.2. Minimum Inhibitory Concentration (MIC)

The minimum inhibitory concentration assay was determined by micro broth dilution method as previously reported by Siddharth and Rai [21]. The serially diluted fraction of extracts with sterile Mueller Hinton broth (Himedia, India) was added to pre coated microbial cultures in 96-well micro titer plates to give a final concentration of 1–3.8 μg/mL. The titer plate was incubated for 24 h at 37 °C. The lowest concentration of extract which completely inhibited the bacterial growth (no turbidity) was considered as MIC. Each test was done in triplicate.

### 2.6. Antioxidant Assays

2.6.1. 2,2-diphenyl-1-picrylhydrazyl Radical Scavenging activity (DPPH)

DPPH radical scavenging activity of crude extracts was examined based on a previously described method by Siddharth and Rai [22]. Crude extracts at varying concentrations (7.81–1000 μg/mL) were reacted with freshly prepared DPPH in methanol (60 mM, Sigma, USA). Reaction mixture was incubated at room temperature for 30 min in the dark prior to the measurement of absorbance at 520 nm. The radical scavenging activity was expressed as IC_50_ (μg/mL). The percentage scavenging of DPPH radicals was computed by the following equation:

DPPH scavenging effect (%) = [(A_o_ − A_1)_/A_o_] × 100 (1)

where, A_o_ = Absorbance of control, A_1_ = Absorbance of crude extracts. Trolox (Sigma) was used as a reference compound whereas methanol was used as blank.

2.6.2. 2,2′-Azino-bis(3-ethylbenzothiazoline-6-sulfonic acid) Radical Scavenging Activity (ABTS)

ABTS radical scavenging activity was performed according to the method developed by Ser et al. [23] with slight modifications. Crude extracts at concentrations (7.81–1000 μg/mL) were mixed with ABTS (Sigma, USA) cation complex and incubated in dark at room temperature for 30 min. The absorbance was measured at 415 nm. The radical scavenging activity was expressed as IC_50_ (μg/mL). The percentage inhibition of ABTS*^+^ radicals were computed by using following equation:

ABTS*^+^ scavenging effect (%) = [(A_o_ − A_1)_/A_o_] × 100 (2)

where, A_o_ = Absorbance of control, A_1_ = Absorbance of crude extracts. Trolox (Sigma) was used as a positive reference.

## 3. Results and Discussion

Soil is among the most productive habitat colonized by a large number of organisms. The rhizosphere soil in the vicinity of plant roots provides essential nutrients in the form of exudates which favors the growth of microbial communities [24]. Soil microbes are a major source of a number of natural products including clinical important antibiotics, immunomodulators, enzyme inhibitors, antioxidants, anti-tumor and anticancer agents. Actinobacteria are abundant in soil, species of *Streptomyces* in particular represent the dominance over other microbes present in soil and play a vital role in recycling of materials and production of important metabolites [25]. Rare actinobacteria genera such as *Nocardia*, *Nocardiopsis*, and *Nocardioides* are also encountered in soils, though their presence is subjected to conditions of soil such as salinity and alkalinity [26]. The rapid emergence of drug resistant pathogens urges the exploration of new niche habitats for the isolation of new microbial species which can contribute to the uncovering of novel, safe, effective, and broad spectrum bioactive compounds [27].

### 3.1. Isolation, Morphological, and Molecular Characterization of Actinobacterial Isolates

**Figure 1:**
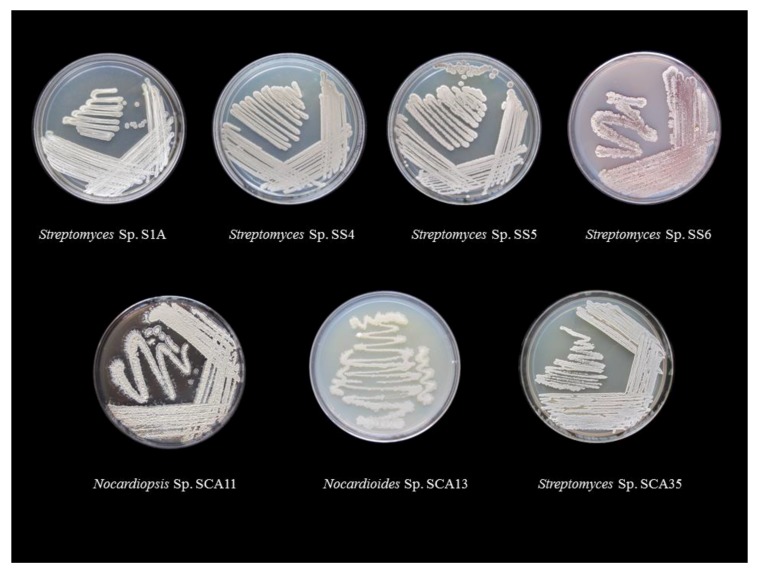
Morphological characterization of actinobacterial isolates on starch casein agar plates.

**Figure 2:**
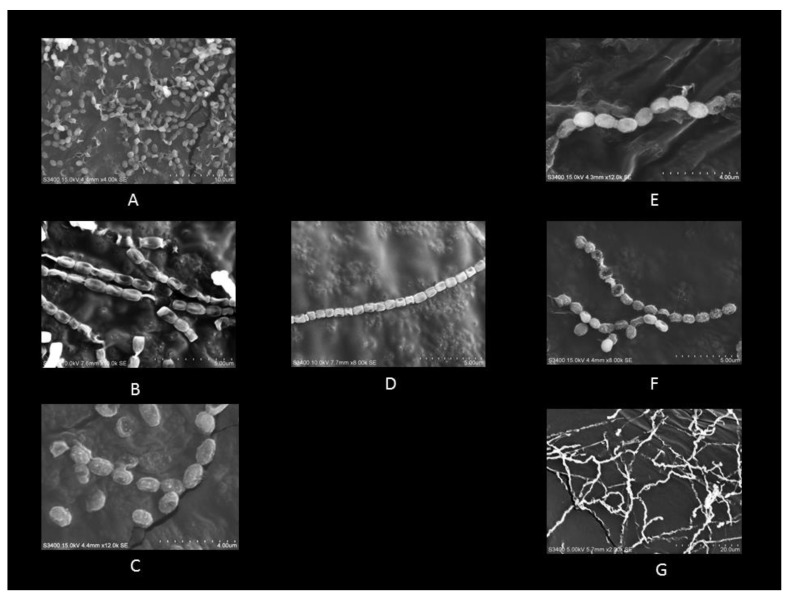
Scanning electron micrograph of strains (**A**) S1A, (**B**) SCA11, (**C**) SS4, (**D**) SS5, (**E**) SS6, (**F**) SCA35, (**G**) SCA13; scale bar represents 5 μm.

**Figure 3:**
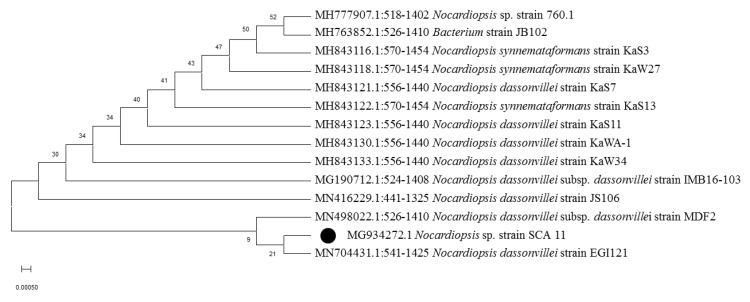
Phylogenetic analysis of isolate *Nocardiopsis* sp. strain SCA11. Neighbor-joining phylogenetic tree showing evolutionary relationship of selected isolate based on 16S r-RNA sequence alignments. Bootstrap values at the nodes indicate collated values based on 1000 resampled datasets. Bar indicates 0.0005 substitutions per nucleotide position.

**Figure 4:**
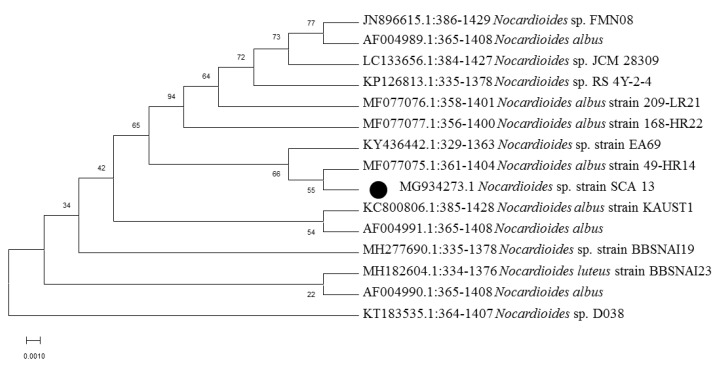
Phylogenetic analysis of isolate *Nocardioides* sp. strain SCA13. Neighbor-joining phylogenetic tree showing evolutionary relationship of selected isolate based on 16S r-RNA sequence alignments. Bootstrap values at the nodes indicate collated values based on 1000 resampleddatasets. Bar indicates 0.001 substitutions per nucleotide position.

**Figure 5:**
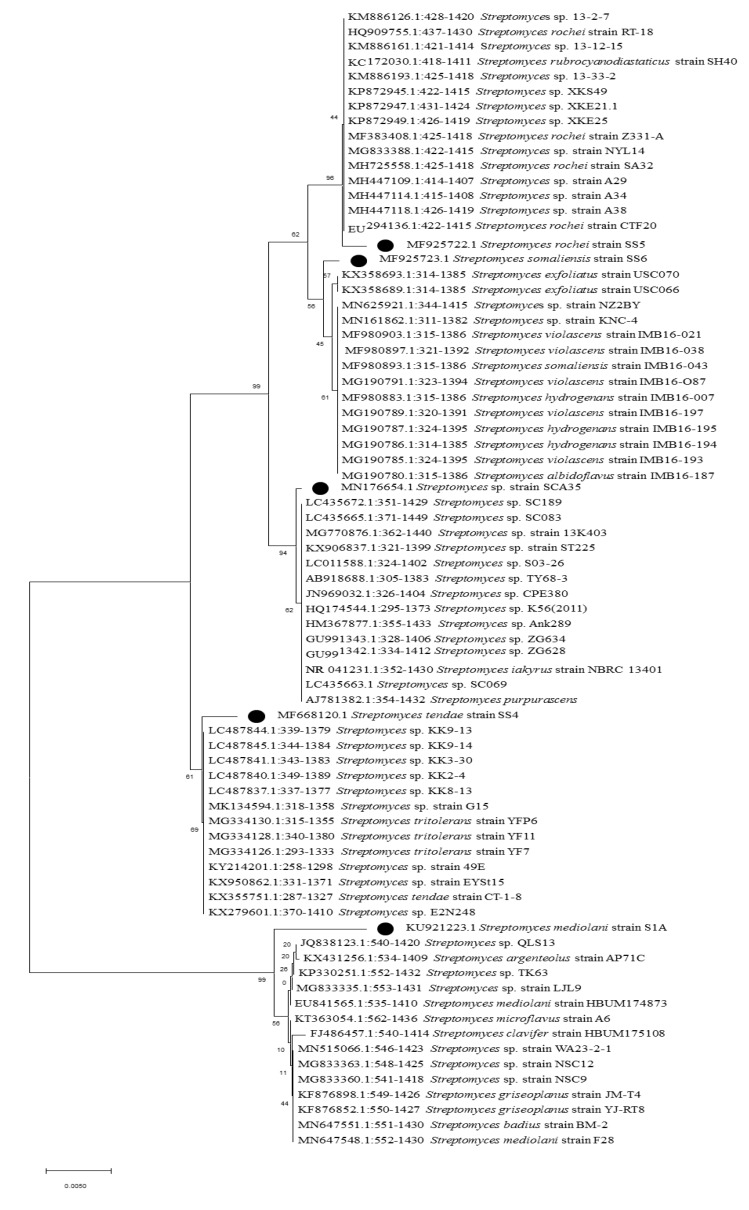
Neighbor-joining phylogenetic tree showing evolutionary relationship between isolates S1A, SS4, SS5, SS6, and SCA35 based on 16S r-RNA sequence alignments. Bootstrap values at the nodes indicate collated values based on 1000 resampled datasets. Bar indicates 0.005 substitutions per nucleotide position.

In this study, from forest soil of Bisle Ghat and Virajpet of Western Ghats region of Karnataka, we targeted the isolation of different genera of actinobacteria in search of new natural products (Table 1). A total of 32 actinobacterial isolates were recovered on Starch casein agar and Actinomycetes isolation agar. Of them, seven isolates grown on starch casein agar medium showing marked antimicrobial activity against test organisms in primary screening were characterized on the basis of cultural, morphological, and molecular characteristics. The colonies of isolates revealed diverse morphological appearances with varied spore color, aerial and substrate mycelium, and colony morphology (Table 2 and Figure 1). Scanning electron microscope examination showed chains of smooth and spiny spores in oval, round, and spiral ornamentation (Figure 2). The molecular identification of isolates by amplification of 16S-rRNA gene was done by using universal primers 27F’ and 1492R’ (Figure S1). In 16S-rRNA sequencing, alignment of the nucleotide sequences of strain S1A, SS4, SS5, SS6, and SCA35 exhibited a similarity of 98.32%, 99.71%, 99.70%, 99.72%, and 99.35% with closely related *Streptomyces* species, respectively. The strain SCA11 was considered to represent a species of the genus *Nocardiopsis*, since it was closely related to *Nocardiopsis* species with 98.99% sequence similarity. The nucleotide sequence of strain SCA13 showed 98.28% similarity with the closely related *Nocardioides* sp. (Table 3). The phylogenetic relatedness of strains with their closely related species obtained by neighbor-joining method is shown in Figure 3, Figure 4 and Figure 5.

### 3.2. Antimicrobial and Antioxidant Potential of Isolates

**Figure 6:**
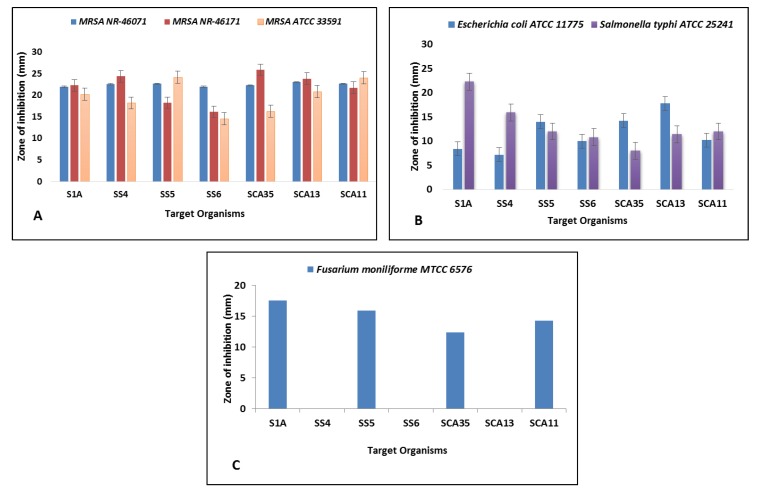
Antimicrobial activity of actinobacterial isolates against (**A**) MRSA ATCC NR 46071, MRSA ATCC NR 46171, and MRSA ATCC 33591 (**B**) *Escherichia coli* ATCC 11775 and *Salmonella typhi* ATCC 25241, (**C**) *Fusarium moniliforme* MTCC 6576.

In this study, all seven isolates showed antibacterial activity against at least one test bacterium. All isolates inhibited MRSA strains significantly (MRSA ATCC 33591, MRSA ATCC NR-46071, and MRSA ATCC 46171), whereas showed less inhibition potential against Gram-negative bacteria *Salmonella typhi* (ATCC 25241) and *Escherichia coli* (ATCC 11775). The isolates namely S1A, SS5, SCA35, and SCA 11 inhibited *Fusarium moniliforme* (MTCC 6576) to a maximum extent (Figure 6). The minimum inhibition concentration (MIC) ranges from 15.62 to 125 μg/mL for MRSA strains, 125–500 μg/mL for Gram-negative bacteria, and 62.5–250 μg/mL for fungi (Table 4). Numerous studies have reported antimicrobial activity of actinobacterial species. Sengupta et al. [28] reported potential antimicrobial activity of three isolates against *Pseudomonas aueroginosa*, *Enterobacter aueroginosa*, *Salmonella typhi*, *Salmonella typhimurium*, *Escherichia coli*, *Bacillus subtilis*, and *Vibrio cholera*. Satheeja and Jebakumar [29] reported isolation of *Streptomcyes* species from a mangrove ecosystem for antibacterial activity against clinical isolates of MRSA, methicillin-susceptible *Staphylococcus aureus* (MSSA), and *Salmonella typhi*. Vu et al. [30] reported antimicrobial activity of *Streptomyces cavourensis* YBQ59 against methicillin-resistant *Staphylococcus aureus* ATCC 33591 and methicillin-resistant *Staphylococcus epidermidis* ATCC 358984. Dashti et al. [31] reported the co-cultivation of *Actinokineospora* sp.EG49 and *Nocardiopsis* sp.RV163 and metabolites produced were tested for antimicrobial activity against the range of pathogens. The bioactive metabolite from *Streptomyces cyaneofuscatus* M-169 showed significant inhibition of Gram-positive bacteria with MIC value of 0.03 μg/mL [32]. It has been shown that actinobacterial isolates from Western Ghats exhibit antimicrobial activity. The *Streptomyces* species from Agumbe [33], *Streptomyces* sp. RAMPP-065 from Kudremukh [34], and *Streptomyces* sp. GOS1 isolated from Western Ghats of Agumbe, Karnataka [35] exhibited remarkable antimicrobial activity. In earlier studies, *Streptomyces* species isolated from Kodachadri were found to possess antifungal activity [33]. Each extract was evaluated for scavenging activity against DPPH and ABTS radicals for antioxidant activity. Crude extract of strain SCA11 showed potent scavenging activity against DPPH radicals with IC_50_ (µg/mL) 30.91 ± 0.25, whereas SCA13 showed remarkable scavenging activity against ABTS radicals with IC_50_ (µg/mL) 37.91 ± 0.17. Trolox as a standard showed significant activity with IC_50_ (µg/mL) 11.07 ± 0.06 and 9.87 ± 0.01 against DPPH and ABTS radicals, respectively (Table 5). Similar studies were carried out for the detection of compounds with antioxidant activity from *Streptomyces* spp., dihydroherbimycin A [36], 5-(2,4-dimethylbenzyl)pyrrolidin-2-one [37]. Ser et al. [38] reported antioxidant activity of pyrrolo[1,2-a]pyrazine-1,4-dione, hexahydro extracted from *Streptomyces mangrovisoli*, a novel *Streptomyces* species isolated from a mangrove forest in Malaysia. Narendhran et al. [39] successfully reported antioxidant potential of phenol, 2,4-bis(1,1-dimethylethyl) in *Streptomyces cavouresis* KUV39 isolated from vermicompost samples. Tian et al. [40] reported antioxidant, antifungal, and antibacterial activity of *p*-Terphenyls isolated from halophilic actinobacteria *Nocardiopsis gilva* YIM 90087. Current findings indicated the bioactive potential of actinobacterial isolates from Western Ghats region of Karnataka. The isolates were found to possess significant antimicrobial activity against Gram-positive MRSA bacteria, Gram-negative bacteria, and fungal pathogens. They also exhibited potent scavenging activity against DPPH and ABTS radicals suggesting their antioxidant potential. It is anticipated that findings of the study will be useful in the discovery of novel species of actinobacteria for a potential source of bioactive compounds from underexplored environments.

## 4. Conclusions

The present study was successful in determining the diversity and bioactive potential of actinobacterial isolates from Western Ghats region of Karnataka. Relatively underexplored forest regions of Western Ghats of Karnataka are found to be promising resources for the discovery of natural bioactive metabolites. The isolates showed significant antimicrobial activity against pathogenic Gram-positive MRSA bacteria, Gram-negative bacteria, and fungi. They were also found to possess antioxidant potential. Our studies encourage the exploration of diverse ecosystems for the isolation of new species for novel and biologically active compounds. Further studies are under progress to purify and characterize the crude extracts that may result in the economic production of bioactive compounds for pharmaceutical applications.

## Figures and Tables

**Table 1:** Actinobacterial isolates and sampling areas in Western Ghats regions of Karnataka, India.

Name	Sample Code	Sampling Area	Latitude (N)	Longitude (E)	Elevation (m)
*Streptomyces* Sp.	S1A	Bisle Ghat, Hassan District	12°71′88.04″	75°68′70.02″	802
*Streptomyces* Sp.	SS4	Bisle Ghat, Hassan District	12°72′00.89″	75°68′41.42″	752
*Streptomyces* Sp.	SS5	Bisle Ghat, Hassan District	12°71′24.30″	75°68′04.74″	710
*Streptomyces* Sp.	SS6	Virajpet, Madikeri District	12°19′75.83″	75°79′52.93″	885
*Streptomyces* Sp.	SCA35	Virajpet, Madikeri District	12°18′83.38″	75°83′06.79″	864
*Nocardiopsis* Sp.	SCA11	Virajpet, Madikeri District	12°21′21.64″	75°80′24.84″	830
*Nocardioides* Sp.	SCA13	Virajpet, Madikeri District	12°18′47.27″	75°76′24.17″	798

**Table 2:** Morphological characteristics of isolated actinobacterial strains.

Isolate	Medium	Diffusible Pigment	Colony Morphology	Aerial Mycelium	Substrate Mycelium
*Streptomyces* Sp. S1A	SCA	None	Powdery	White	Cream
*Streptomyces* Sp. SS4	SCA	None	Cottony	Dark Grey	Grey
*Streptomyces* Sp. SS5	SCA	None	Cottony	Grey	Cream
*Streptomyces* Sp. SS6	SCA	Pink	Rough	Pale red	Pink
*Streptomyces* Sp. SCA35	SCA	None	Powdery	White	Cream
*Nocardiopsis* Sp. SCA11	SCA	None	Powdery	Cream	Light brown
*Nocardioides* Sp. SCA13	SCA	None	Raised	White	White

**Table 3:** Molecular identification (based on 16S rRNA amplification) of actinobacterial strains isolated from Western Ghats.

Source	Organism	GenBank Accession No	% Similarity
Soil	*Streptomyces* Sp. S1A	KU921223	98.32%
Soil	*Streptomyces* Sp. SS4	MF668120	99.71%
Soil	*Streptomyces* Sp. SS5	MF925722	99.70%
Soil	*Streptomyces* Sp. SS6	MF925723	99.72%
Soil	*Streptomyces* Sp. SCA35	MN176654	99.35%
Soil	*Nocardiopsis* Sp. SCA11	MG934272	98.99%
Soil	*Nocardioides* Sp. SCA13	MG934273	98.28%

**Table 4:** Minimum inhibitory concentration (MIC in µg/mL) of actinobacterial isolates against pathogenic test organisms.

Organisms	Minimum Inhibition Concentration (µg/mL)					
	Test Organisms					
	MRSA ATCC NR-46071	MRSA ATCC NR-46171	MRSA ATCC 33591	*Salmonella typhi* ATCC 25241	*Escherichia coli* ATCC 11775	*Fusarium moniliforme* MTCC 6576
*Streptomyces* Sp. S1A	15.62	15.62	62.5	125	>500	62.5
*Streptomyces* Sp. SS4	62.5	31.25	125	250	>500	-
*Streptomyces* Sp. SS5	31.25	125	15.62	250	250	125
*Streptomyces* Sp. SS6	31.25	62.5	62.5	250	125	-
*Streptomyces* Sp. SCA35	62.5	15.62	125	>500	125	250
*Nocardiopsis* Sp. SCA11	31.25	15.62	31.25	250	>500	125
*Nocardioides* Sp. SCA13	62.5	62.5	125	250	125	-

**Table 5:** Comparison of IC_50_ (µg/mL) of crude extracts and trolox for DPPH and ABTS radical scavenging activity.

Isolates	IC_50_ (µg/mL) Crude Extracts	
	DPPH	ABTS
*Streptomyces* Sp. S1A	189.40 ± 0.12	156.81 ± 0.06
*Streptomyces* Sp. SS4	98.29 ± 0.32	123.48 ± 0.13
*Streptomyces* Sp. SS5	86.45 ± 0.04	114.87 ± 0.29
*Streptomyces* Sp. SS6	114.15 ± 0.03	164.04 ± 0.07
*Streptomyces* Sp. SCA35	65.86 ± 0.49	49.11 ± 0.73
*Nocardiopsis* Sp. SCA11	30.91 ± 0.25	48.24 ± 0.30
*Nocardioides* Sp. SCA13	42.30 ± 0.10	37.91 ± 0.17
**Trolox (Standard)**	11.07 ± 0.06	9.87 ± 0.01

## Supplementary Materials

Supplementary materials can be found at https://www.mdpi.com/2076-2607/8/2/225/s1.
