# Supplementary material for: Diversity and Bioactive Potential of Actinobacteria from Unexplored Regions of Western Ghats, India
Source: Microorganisms. 2020 Feb 7;8(2):225. doi: 10.3390/microorganisms8020225 (PMC7074718; doi:10.3390/microorganisms8020225)
Supplement: Supplementary file 1 [file microorganisms-08-00225-s001.pdf]

Diversity and Bioactive Potential of Actinobacteria from unexplored regions of Western Ghats, India

Saket Siddharth, Ravishankar Rai V, Joachim Wink and Michael Steinert

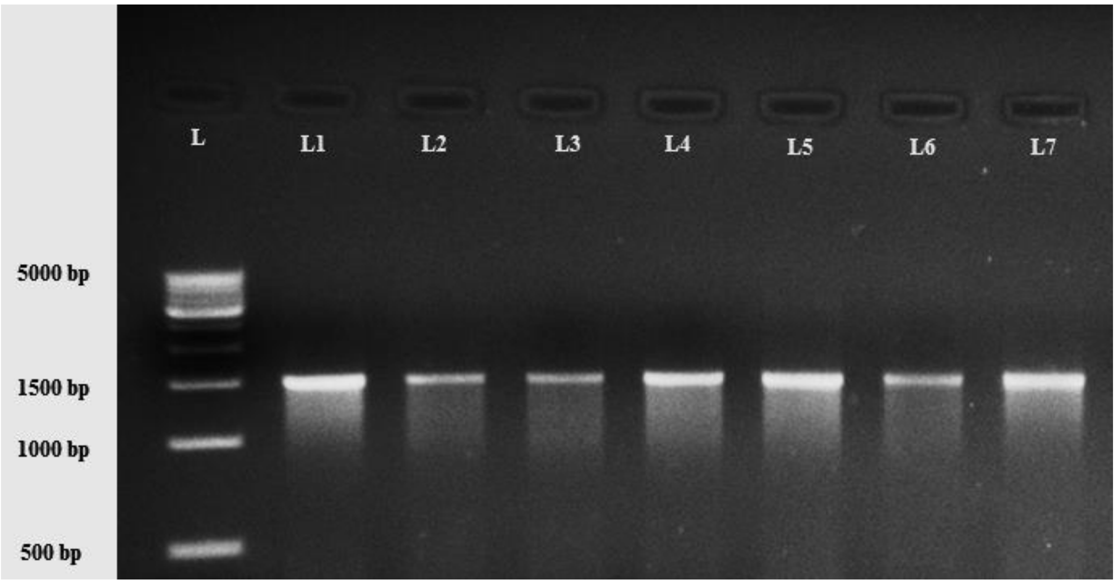

Figure 1. Agarose gel electrophoresis (1.5%) of PCR amplified products of 16S-rRNA genes. ***L***: 500bp DNA ladder, ***L1***: Amplified PCR product of strain S1A, ***L2***: Amplified PCR product of strain SS4, ***L3***: Amplified PCR product of strain SS5, ***L4***: Amplified PCR product of strain SS6, ***L5***: Amplified PCR product of strain SCA35, ***L6***: Amplified PCR product of strain SCA11, ***L7***: Amplified PCR product of strain SCA13.
